# Do Domestic Dogs Learn Words Based on Humans’ Referential Behaviour?

**DOI:** 10.1371/journal.pone.0091014

**Published:** 2014-03-19

**Authors:** Sebastian Tempelmann, Juliane Kaminski, Michael Tomasello

**Affiliations:** 1 Department of Developmental and Comparative Psychology, Max Planck Institute for Evolutionary Anthropology, Leipzig, Germany; 2 Department of Psychology, University of Portsmouth, Portsmouth, United Kingdom; 3 University of Applied Sciences and Arts Northwestern Switzerland, Liestal, Switzerland; Centre for Coevolution of Biology & Culture, University of Durham, United Kingdom

## Abstract

Some domestic dogs learn to comprehend human words, although the nature and basis of this learning is unknown. In the studies presented here we investigated whether dogs learn words through an understanding of referential actions by humans rather than simple association. In three studies, each modelled on a study conducted with human infants, we confronted four word-experienced dogs with situations involving no spatial-temporal contiguity between the word and the referent; the only available cues were referential actions displaced in time from exposure to their referents. We found that no dogs were able to reliably link an object with a label based on social-pragmatic cues alone in all the tests. However, one dog did show skills in some tests, possibly indicating an ability to learn based on social-pragmatic cues.

## Introduction

Different animal species can be taught to respond in particular ways to human words and other arbitrary symbols (e.g., African Grey Parrots: [1]; dolphins: [2]; chimpanzees and bonobos: [3], [4]; sea lions: [5]. However, the degree to which these behaviours resemble human language learning and comprehension is a difficult issue.

A species of particular interest in this regard is the domestic dog, as dogs have evolved during the domestication process to use human communication in special ways [6], [7]. In a recent study Kaminski, Call, and Fischer (2004) investigated the ability of a Border Collie named Rico to link words with the objects to which they referred (see also [8] for a similar study with another Border Collie called Chaser). In this study the authors found that Rico was indeed capable of linking roughly 200 labels to their referents. Moreover, Rico was found to be able to learn new word-object links through exclusion and, to some degree, to store this knowledge in memory [9] see also [8]. This performance is comparable to the fast mapping abilities only previously reported for human children [10]–[12]. However, the question remains as to how Rico, or any other dog, actually understands the word-object relation [13], [14]. One question is whether a dog would be able to learn a word-object relation by using the referential behaviour of a human speaker, which Rico’s behaviour would suggest, or whether dogs are generally just able to form those links by blind association based on spatial-temporal contiguity alone.

Children from the age of 18 months are able to infer the meaning of words from certain social-pragmatic cues (e.g., [15]–[17]. This enables them to form word-object relations in ways that go beyond simply associating simultaneously present visual and auditory stimuli. Children are thus able to use the referential intent behind another individual’s utterances to form word-object relations even without spatial-temporal contiguity between the word and the referent, such as when an adult looks into an opaque bucket and names an object which the child does not see until a few moments later (e.g., [18]–[20]; see [21] for a review). Understanding referential intentions independent of spatial-temporal contiguity is often seen as crucial for the development of a communication system consisting of arbitrary symbols, like human language (e.g., [22]).

In recent years more and more evidence has emerged that dogs are especially adapted to use human forms of communication, especially human gestural communication. They seem to use human referential behaviour (especially pointing, the archetype of non-verbal reference; [23]) in a very flexible manner that cannot be explained by low-level explanations such as local enhancement (for an overview see [24]). That this seems to be a special adaptation to human societies is supported by several facts. First, in their ability to use human gestures, dogs outperform humans’ closest relative, the chimpanzee [25]–[27]. Also, dogs’ skills in this domain do not seem to represent a general canid skill, since the dog’s closest living relative, the wolf, is not as skilful at pointing as dogs [26], [28]. Finally, dogs’ skills do not seem to be the result of extensive individual learning to follow cues during their ontogeny, since puppies from a very young age already follow certain gestures [26], [29], [30]. These findings suggest that a dog’s skill in comprehending human communicative behaviour is a species-specific adaptation for interacting effectively within human society [7], [28], developed during the process of domestication (see [31], [32] for a discussion of this hypothesis).

A recent study provides evidence that a dog’s understanding of gestures is more flexible than previously thought. Kaminski, Schulz, and Tomasello (2012) confronted dogs with a situation where a human intentionally gestured towards one of two cups in order to indicate the location where a reward was hidden (e.g., by pointing to it). In a control condition the human produced certain unintended behaviours, directed at the target, which mirrored to some extent the communicative gesture (for example, the human held her arm in an outstretched position to check the time on her watch). The dogs in this study followed the intended communicative gesture but ignored the unintended behaviours [33]. In another study [34] dogs were confronted with a task in which they had to infer the intended referent of a human’s communicative act via iconic signs – a totally novel situation for them. All dogs were to some degree able to use the iconic signs, in most cases from the beginning and without learning. Taken together these results indicate that dogs may comprehend important aspects of human communicative intentions.

However, so far we do not know how flexible this understanding is and whether it relates to dogs’ ability to learn “words” (e.g., labels for objects). In the following studies we examined whether dogs, specially trained to retrieve objects by their names, were able to learn the labels for new objects in contexts during which there was no spatial-temporal contiguity between the acoustic word and the visual referent. Instead the dogs had to use the speaker’s referential behaviour (e.g., pointing, gaze alternation) to infer the referent, which was out of their view. In the first study (modelled on [19]), one object was hidden in each of three buckets. Then the human labelled one of the objects, still out of view of the dog, by repeatedly directing her attention to the object in the bucket. After labelling was complete, all objects were tipped out such that they were in full view of the dog. However, now the human sat still and did not refer to any of them. In the second study modelled on [15], we confronted dogs with a situation in which they had to inhibit any association of the word they heard with an object they saw, as the human was in fact referring to a different object which only she, the human, could see on her side of a barrier.

One constraint with the current study is that only four dogs could be tested. This is because dogs with the skills needed for this kind of study are extremely rare. The four dogs we worked with have been taught numerous words by their owners – that is, taught to fetch numerous different objects by name and have been shown to be able to reliably do so under experimental conditions [35], [36].

## Experiment 1

### Methods

The owners of the dogs were informed about each aspect of the study and gave their permission for the different studies. The studies presented here are not invasive. IRB approval was not necessary for this kind of study because no special permission for use of animals (dogs) in such socio-cognitive studies is required in Germany, Austria or Switzerland. All procedures were performed in full accordance with German, Austrian or Swiss legal regulations and the guidelines for the treatment of animals in behavioural research and teaching of the Association for the Study of Animal Behaviour (ASAB).

The general procedure was based on studies conducted by Tomasello and colleagues [19], [20] with 18- and 24-month-old children. The main goal of this first experiment was to see whether dogs that were particularly skilful in learning labels for objects would be able to learn a new label in non-associative contexts. To test this we confronted dogs with a situation in which there was no spatial-temporal contiguity between stimuli (seeing the object and hearing its label), but rather the cues were referential-intentional cues. To evaluate different complexity factors, when pre-testing this study we ran a pilot phase during which we focussed on 3 slightly different versions of the experiment.

#### Subjects

The subjects were 4 domestic dogs (Betsy, a 4-year-old female, Paddy, a 5-year-old male, Joey, a 4-year-old male and Arco, a 5-year-old-male. All dogs were Border Collies.), who had received special training in fetching items according to their labels and were able to do so reliably under experimental conditions (for Betsy and Paddy see [35], [36], for Arco and Joey: unpublished data ). The procedure for testing these abilities in the different dogs was based on the original procedure developed by Kaminski et al. 2004 [11]. All four dogs knew more than 30 objects by name, which was the threshold for including them in this study. However, the dogs’ label pools varied between 35 labels (Arco) and 230 labels (Betsy).

The owners of the dogs reported that the normal routine for learning new object-label links involved the dogs interacting with the objects whilst the owners constantly repeated the labels. All four dogs live as pets with their owners.

#### Experimental set-up and general procedure

**Figure 1:**
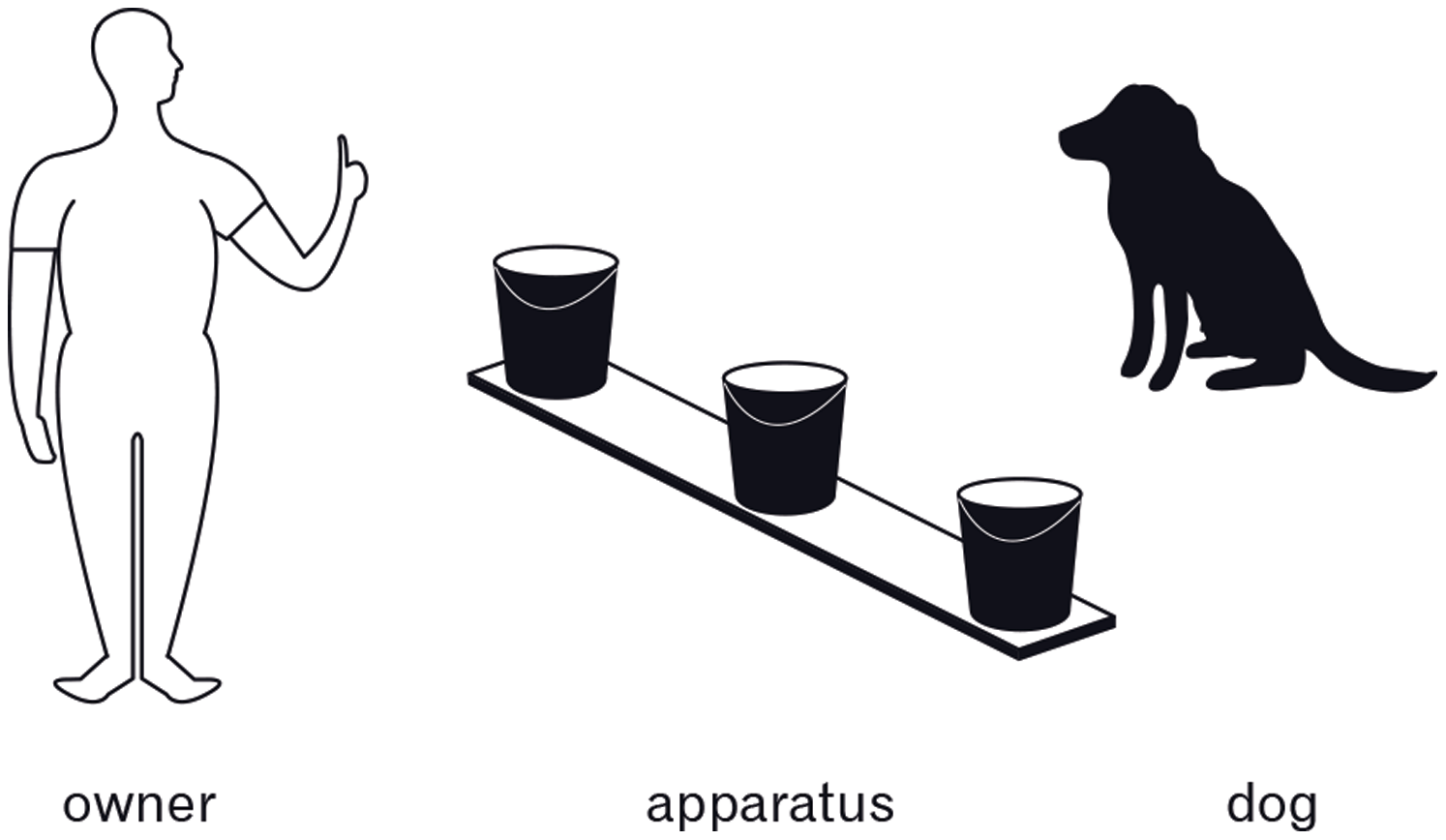
Experimental set up during the naming phase of Experiment 1.

The apparatus consisted of three identical opaque plastic buckets (37 cm deep, 32 cm in diameter) fixed on a longitudinal wooden slat (see figure 1). This allowed the buckets to be tilted simultaneously by rotating the wooden slat. The distance between the buckets was 48 cm. The objects used were mainly dog and children’s toys, all of which were unfamiliar to the test dogs.

The basic procedure was identical for all dogs. Both the experimenter (E) and the owner (O) were present during testing. Testing took place in two adjacent rooms in the dog owner’s apartment. For each trial a new set of 3 toys was randomly selected from an array of toys which were similar in size and material. Each trial consisted of two phases. The label for a new toy was introduced to the dog during the initial naming phase. The novel labels were selected on the basis of their being as unfamiliar and as phonologically distinctive from the previously known labels as possible (see table 1 for examples). This means that we tried to avoid introducing new labels which matched labels the dogs already knew (e.g., introducing a new label ‘pig’ when the dog already owned an object labelled ‘stick’). This phase was followed by a retrieval phase during which the owner asked the dog to fetch the object from an adjacent room and from three different objects to see whether the dog had learnt the new labels.

**Table 1:** Examples of labels introduced during Experiments 1 and 2.

Subject	Study	Label
**Paddy**	**1**	Gysi, Hot Dog, Gluehbirne, Bruno, Lumpi, Regenbogenhantel, Beetle,Quadratschaedel, Spirale, Muente
	**2**	Heini, Spektrum, Snutig, Boomer, Harlikin, Moby Dick, Kilogramm, Petzi, Topolino, Neopren, Michael Jackson
**Betsy**	**1**	Niederoesterreich, Stechapfel, Alfons, Schrubka, Titifax, Columbus, Aristotoles, Klecksi, Propeller, Tantalus
	**2**	Washington, Arabella, August, Miranda, Torpedo, Schokodrop, Heinzelmaennchen, Ikea, Knautschli, Rabe

During the naming phase O waited with the dog in the starting room, while E set up the apparatus in the adjacent second room. E placed one object in each of the three buckets, one of which was the target object. The position of the target toy was counterbalanced across trials. The hiding process could not be observed by the dog. After the hiding process was finished the owner and the dog entered the testing room while E remained sitting still in a predetermined position, about 2.5 metres behind the buckets, facing the middle bucket. The dog could not see the objects in the buckets at any time while entering. E then took over handling the dog from O. While E held the dog on a lead the owner sat in a predetermined position in the centre of the apparatus facing the dog.

#### Pilot phase

During an initial pilot phase, the naming procedure was different for each dog. This was to see which procedure could be successful. In order to reduce the chances of learning during this phase, not every procedure was used with every dog.

#### Paddy

During naming of the target object, the owner pointed at the target whilst saying, for example, “What a nice xy!”. The object remained in the bucket throughout, hidden from the dog’s view. The naming of the new toy was repeated up to seven times to ensure that the dog had enough exposure to the label. The owner ignored the other two buckets and hence the other two objects. After naming was finished, the owner remained still with a neutral body posture.

#### Joey & Arco

The naming period was similar to the above, although now instead of the owner just focussing on the target object and ignoring the contents of the other two buckets, the buckets containing the non-target objects were onomatopoetically treated by saying, “Ohh, ahh” (no words were used). The owner directed her attention to each bucket one after the other, always starting with the one on the left. The duration of attention towards the bucket as well as intensity and intonation of vocalizations was similar for both target and non-target buckets.

#### Betsy

Again the naming procedure was similar, but now the owner’s behaviour towards the other two buckets was identical to that towards the bucket containing the target object; however, instead of labelling the objects inside, the owner simply said “Oh, look, what a nice toy inside!” without stating a label. The owner directed her attention towards each bucket, one after the other, always starting with the one on the left. The duration of attention towards the bucket as well as intensity and intonation of vocalizations was similar for both target and non-target buckets.

After the naming was finished, O grabbed the slat with her hands and simultaneously emptied the contents of all three buckets by tilting the apparatus towards the dog. The dog remained in its position next to E and was not allowed to touch the toys. Then O put the toys back into the buckets from left to right and brought the apparatus back into its upright position. The entire naming procedure was repeated three times before the requesting phase started. In this phase E placed all three toys (lined up in a straight row, with the position of the target object randomized) in the adjacent room while the owner and the dog remained in the initial room. Having positioned the objects E joined the owner and the dog in the other room and the owner then requested that the dog retrieve the target toy using the new label introduced during step 1 (e.g., “Bring me the xy!”). The dog could not see the owner or E while searching for the requested item. If the dog retrieved the correct item the owner rewarded the dog with play and/or food. In cases in which the dog retrieved an incorrect object, i.e. an object other than the target object, the owner reiterated her command and let the dog search for the target once more. After that the next trial started.

Dogs received a total of 24 trials of the respective version of this pilot study and trials were conducted over six days, with four trials per day. A total of 72 toys were randomly selected for each dog. Toys were all similar in size and material and were randomly grouped into 24 sets of three toys each.

#### Main experiment

After an initial analysis revealed that only Paddy performed above chance (with chance being 0.33, see a more detailed description of all results below) we conducted the main experiment using the naming procedure used with Paddy on all other dogs. This main experiment was conducted several weeks after the initial study to ensure that there were no major carry-over effects.

The procedure and number of trials were exactly the same as described for Paddy in the pilot phase. The subjects were Betsy, Joey and Arco.

### Results

#### Statistical procedure

For statistical analysis of each dog’s overall performance (both attempts for a given trial) we used a Monte-Carlo simulation procedure [37]. For this we simulated trials during which an individual randomly picked toys from a set of available toys, one after the other and without replacement (i.e. each choice reduced the number of toys available for the next choice), until it retrieved the target toy (1 out of N). The number of toys available at the beginning of a trial was the three objects presented during the naming phase. We then ran as many trials as the original subject(s), and calculated the average number of attempts a subject needed to retrieve the target object (if at all). We then repeated the entire process 10,000 times, in this way generating the expected frequency distribution for the average number of attempts after which the subject would bring the target toys if choosing randomly. The real subjects, if they were using the labels of the toys, should bring the target toy on average after fewer attempts than in this expected (chance) distribution. Significance was determined by calculating the proportion of simulations in which the average number of attempts to fetch the target object was as small as or smaller than for the real subject. We also assessed each dog’s performances on the first attempts of each trial, using a straightforward binomial procedure.

In the pilot phase chance expectation would be 0.33, corresponding to the three objects presented. Paddy retrieved the target object in 22 of the 24 trials (Monte-Carlo test, N simulations = 10.000, *P = *0.0073; see table 2). Reducing the data to only his first attempts in each trial, he retrieved the target object 12 times, which is not significant (binomial test, *P* = 0.126 (2-tailed)). None of the other dogs performed above chance with either statistical method (see table 2 for more detailed information).

**Table 2:** Dogs’ performance in Experiment 1 (Monte Carlo test N simulations = 10000; binomial test, chance level = .33).

	First run	Second run		
Subject	Correct retrievals overall	Correct retrievals 1st attempt	correct retrievals overall	Correct retrievals 1st attempt
	(Monte Carlo simulations)	(binomial test)	(Monte Carlo simulations)	(binomial test)
**Paddy**	22 (p = .0073)	12 (p = .063)		
**Arco**	15 (p = .4215)	11 (p = .132)	19 (p = .4452)	6 (p = .275)
**Joey**	17 (p = .3002)	10 (p = .243	19 (p = .0826)	11 (p = .132)
**Betsy**	17 (p = .1895)	11 (p = .132)	16 (p = .4417)	9 (p = .392)

In the main experiment all dogs received the naming procedure with which Paddy had been successful. Here the owner ignored the non-target objects and exclusively directed her behaviour towards the bucket which contained the target object. None of the other dogs performed above chance with this procedure (see table 2).

### Discussion

This study has two main findings: First, as seen in literature on animals but also in the developmental literature there seem to be individual differences between subjects. While three dogs were not at all able to use any aspect of the referential act to identify the target object, one dog was able to use the referential act to identify the task, even though the success rate was not overwhelming even in this dog. This suggests that, while this skill could indeed be within the species’ range, overall the task seemed fairly demanding for the dogs. Even for the one dog, which was possibly able to link the label and the object in the current situation, his performance might have been simply due to a delayed association process resulting from a local enhancement of one of the buckets and, therefore, its contents. It could be that for the dog, the human’s deictic behaviour did not refer to the object itself but rather to the location, which was in consequence enhanced and might have subsequently enhanced the object. In addition, assuming the dog understood that the referential action of the human was directed towards the object, the question remained whether he linked the word with the object or just retrieved the object the human had previously referred to.

In order to address this question we conducted a second experiment with Paddy only since he was the only dog to have been successful in Experiment 1.

## Experiment 2

In Experiment 1 Paddy showed that he was able to infer from the human’s behaviour which object the human wanted him to fetch. However, this does not necessarily mean that Paddy was able to link the object with the label he had heard. The following experiment was conducted to further test this. Now Paddy was presented with two pairs of objects one after the other, with one object from each pair being labelled. During the experimental phase he then had to decide between both labelled objects upon hearing the command to fetch one of the two. The labelling procedure for each target object was identical to the procedure used with Paddy in the previous study.

## Methods

### 

#### Subject

Paddy (see Experiment 1).

#### Experimental set-up and general procedure

A board was used with two opaque buckets (the same buckets used in Experiment 1) attached to it, fixed on a longitudinal wooden slat. The distance between the buckets was 48 cm. A rack was also used with an opaque and drawable curtain. As in Experiment 1 we used children’s and dog toys that were unfamiliar to the dog.

Each trial consisted of a naming phase during which the labels for target A and target B were introduced. This phase was followed by a requesting phase during which one object was requested using the label introduced just previously. During the naming phase the labels (for target A and target B) were introduced while each target object was paired with a distracter (distracter A and distracter B). The general procedure was similar to Experiment 1. The owner (O) sat behind the apparatus, with the dog sitting opposite and a curtain covering the owner during the initial handling of the objects. The owner placed both objects of a given pair into the buckets (starting with the left-hand bucket). Then she opened the curtain and labelled the target object repeatedly by looking back and forth between the respective bucket and the dog. Labelling was repeated up to 4 times.

Afterwards the owner dumped both objects on the floor without giving any directional cues whatsoever to any of the objects. After 5 seconds O closed the curtain again and continued by placing the next pair of objects (A or B respectively) into the buckets; the whole procedure was repeated. Each pair of objects was presented 4 times to the dog, always alternating between pair A and pair B. The order of presentation in the labelling phase (target A and B and the respective position) and the positions of the objects during the requesting phase were semi-randomized, with the stipulation that the same pair could not appear in more than 2 consecutive trials, and counterbalanced.

In the requesting phase all four objects of both pairs were placed in an adjacent room. Then the dog was requested to “Fetch target A” or “Fetch target B” respectively. If the dog returned with the correct object he was rewarded. If the dog returned with the incorrect object, the owner took the object, put it away and the trial ended. If the dog returned with no object, upon her return the command was repeated and the dog was again send to the other room.

Paddy received 24 trials during which a total of 48 labels were introduced. After 13 trials Paddy showed signs of fatigue (e.g., constantly lying down, no response to commands) during the naming phase, which is why we reduced the number of introductions from 4 two 2 per pair.

### Results

Paddy retrieved the correct object in only 7 out of 24 trials. Assuming a chance level of 50% (corresponding to the two objects that had been labelled) the dog’s performance was close to being below chance level (binomial test, *P* = 0.064, 2-tailed). Paddy retrieved a labelled object, irrespective of whether it was the correct one or not, in eleven out of 24 cases (binomial test, *P = *0.839, 2-tailed). When target A had been requested Paddy correctly retrieved it in 6 out of 12 cases, but retrieved target B correctly in only 1 out of 12 cases (χ^2^ = 5.042, df = 1, *P* = 0.025, 2-tailed). When he brought an incorrect object, in 4 cases out of 17 this was the labelled non-target object, which was significantly below chance expectation (binomial test, *P* = 0.007, 2-tailed).

### Discussion

Paddy’s behaviour in this study clearly shows that he was struggling to link the word he heard with the specific object, as he did not differentiate between the two different objects on the basis of the newly learned labels. The comparison between both target pairs suggests that Paddy memorized the labels for the target pair which was introduced first better than those for the pair introduced later. This could indicate that memorizing all labels was simply too demanding. However, with the labels introduced first in each trial Paddy’s success rate in fetching the target was also not above chance level, indicating that he did not learn the labels during the course of events. As Paddy’s success rate with retrieving any labelled object (irrespective of whether it was the correct one or not) was not above chance either, this is an indication that in this experiment Paddy failed to use any of the human’s behaviour as an indication that these objects were relevant. Taken together this evidence supports the assumption that the current experiment was too demanding for Paddy.

## Experiment 3

Experiment 1 indicated that one dog might be able to link a word with a particular object on the basis of social-communicative cues given by a human. As the second experiment was probably cognitively too difficult, we introduced a third experiment, modelled on Baldwin, 1991, during which the dogs had visual access to an object, but in some conditions that object was not the referent of the human’s communication. To understand which object the owner had in fact been referring to, in some trials the dogs had to inhibit any association of the label with the object in their view and instead link it with an object which was not in view – as it was on the human’s side of an opaque barrier. Therefore, associative learning based on the spatial-temporal contiguity would lead to an actual error.

### Methods

#### Subjects

The subjects were the same four domestic dogs who participated in Experiment 1.

#### Experimental set-up and general procedure

**Figure 2:**
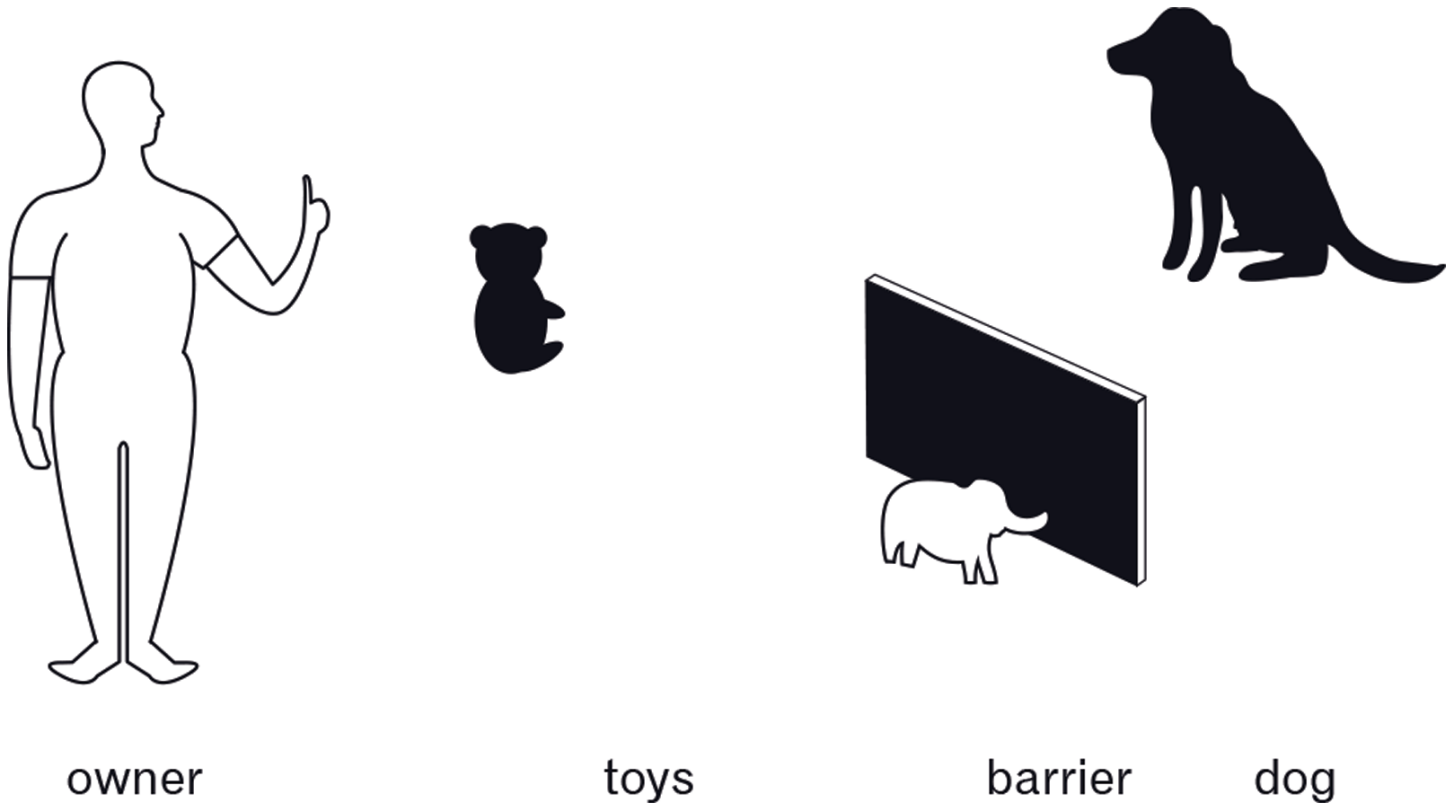
Experimental setting of Experiment 3.

The apparatus consisted of an opaque barrier made of stiff cardboard (33 cm×51 cm) which occluded one of two objects. Again, the objects were again dog and children’s toys unfamiliar to the test dogs. During the experimental phase one object was placed behind the barrier while one lay out in the open in full view. The distance between the objects was 45 cm (see figure 2).

As in Experiment 1 the experimenter (E) and the owner participated in the testing procedure. The testing took place in the same rooms as in Experiment 1. A total of 56 toys were randomly grouped into 28 sets of 2 toys. The stipulation for finding object pairs was that objects had to be similar in size and material. In the experimental phase each dog received a total of 28 trials. Dogs were tested on seven different days, with four trials a day. A new set of two toys was used for each trial. As in Experiment 1 the procedure of the experimental phase consisted of an initial naming phase followed by a requesting phase. Again novel labels were selected on the basis that they were unfamiliar to the dogs and phonologically distinctive from the previously known labels (see table 1 for examples).

Before the naming phase, the owner waited with the dog in one room while E set up the apparatus in the adjacent second room. He placed two of the toys on the floor and set up the barrier in front of one of them. The hiding process could not be observed by the dog. After the hiding process E sat in a predetermined position, approximately 2.5 metres from the apparatus, facing its centre. Then the owner and the dog entered the testing room in such a way that the dog could briefly see both toys. This was done to ensure that the dog was aware that an object had been placed behind the barrier. The owner guided the dog to where E was sitting so that E could hold on to it: From this position, the dog was no longer able to see the object behind the barrier. The owner sat in a predetermined position behind both objects, opposite the dog.

During the naming phase, the owner pointed and gazed at one of the toys and named it up to 7 times with a word unfamiliar to the dog (e.g., “Look, that is the xy.”). Throughout the naming process, the dog remained in its position next to E and was not allowed to touch the toys. In half of the trials the owner directed her attention to the object visible to both her and the dog. In the other half of the trials, the owner directed her attention to the object which was only visible to her and not the dog. The position of the barrier as well as the type of trial was randomized and counterbalanced across trials.

After the naming process was complete the owner came to E’s side and held on to the dog while E grabbed both toys (such that they were unseen by the dog) and placed them in the adjacent room. The experimenter then returned to the owner and dog in the first room and the owner now requested that the dog fetch an object using the label introduced in the naming phase. If the dog retrieved the correct object it was rewarded with food and/or play. The position of the target object was counterbalanced and randomized. To analyse the data we looked at overall performance as well as both conditions separately.

### Results

In this experiment chance expectation would be 0.5, corresponding to the two objects presented. Overall, Paddy retrieved the target object in 18 out of 28 trials (binomial test, *P = *0.185, 2-tailed). This is irrespective of condition (target in the open vs. target behind barrier) as Paddy chose randomly regardless of whether the target was out in the open or behind the barrier (9 out of 14 trials; binomial test, *P = *0.424, 2-tailed). However, Paddy may have learned a strategy during the study because if we compare the last 12 trials of the study to the first 12 trials it seems that Paddy learned to select the target (first 12 trials: correct retrieval in 6 out of 12 times, binomial test, *P = *1.0; last 12 trials: correct retrieval in 10 out of 12 times, binomial test, *P = *0.039, 2-tailed). Note that it was not the case that Paddy was using an associative strategy in the first 12 trials, which he then switched. In the first 12 trials he chose randomly; so in no phase of the experiment did he use an associative strategy. The most plausible hypothesis is that with more experience, Paddy was able to deal with the unusual context and to learn the label for the object on the basis of the referential cues in the situation.

The behaviour of the second dog, Betsy, looked somewhat different. Betsy correctly retrieved the named toy in 17 out of 28 trials (binomial test, *P = *0.345, 2-tailed). In the condition where the owner named the occluded object Betsy retrieved the target object in 6 out of 14 trials (binomial test, *P = *0.791, 2-tailed). However, in the condition where the object which was out in the open was named Betsy retrieved the target object in 11 out of 14 trials, which was marginally significant (binomial test, *P* = 0.057, 2-tailed). In contrast to Paddy she did not improve her performance over trials (in both the first and the last 12 trials she correctly retrieved 7 objects, binomial test, *P = *0.774, 2-tailed). However, she did not have a general preference for the object in the open, as overall she chose it in 19 out of 28 cases (binomial test, *P = *0.087). This speaks against a strategy of generally mapping the word to the visible object, and thus against a word-object mapping based on the spatial-temporal contiguity of the two stimuli. Instead it seems to suggest that the failures are due to the complexity of the task. Betsy’s better performance when the object left in the open was named might therefore be due to the less challenging requirements in this condition, as the target object is more salient and memorable.

The performance of Joey and Arco is comparable to that of Betsy, with the difference that their performance did not even reach a level close to significance. Joey retrieved the correct object in eleven out of 28 cases (binomial test, *P* = 0.345, 2-tailed) and Arco in 15 out of 28 trials (binomial test, *P* = 0.851, 2-tailed).

When asked to retrieve an object which was named in the open, Joey chose the correct one in four out of 14 cases (binomial test, *P = *0.180, 2-tailed) and Arco in nine out of 14 cases (binomial test, *P = *0.424, 2-tailed). Joey correctly retrieved the object which was named when placed behind the barrier in seven out of 14 cases (binomial test, *P = *1.000, 2-tailed), and Arco in six out of 14 cases (binomial test, *P = *0.791, 2-tailed). Furthermore, both dogs did not improve their performance, as it was identical in the first and in the last twelve trials; Joey retrieved the correct object four times (binomial test, *P = *0.388, 2-tailed), and Arco seven times (binomial test, *P = *0.774, 2-tailed).

Like Betsy they did not have a general preference for the object in the open. Arco chose the object which was presented in the open during the naming phase in 17 out of 28 trials, and Joey in eleven out of 28 (binomial test, for both dogs *P = *0.345, 2-tailed).

### Discussion

These results show that three of the tested dogs made no object-word links in any of the conditions. Strangely, they did not even make object-word links in those conditions where there was the potential for associative learning. This could suggest that for most of the tested dogs the situation was cognitively too demanding. One exception was Paddy, who showed some ability to link the label with the object based on E’s referential behaviour when it competed with simple association – though he needed some experience with the paradigm to be able to do so as this ability only appeared towards the end of the study.

## General Discussion

In sum, the dogs in the current studies were not able to make object-word links in the different contexts we provided. This could be evidence that making object-word links outside an associative context is simply too difficult a task for dogs. Strangely, the dogs did not make object-word links when the condition gave room for simple association either, indicating that the paradigms were probably generally too demanding for the dogs. One exception was Paddy, who performed well in parts of the studies presented.

In the first experiment phase of Experiment 1 Paddy seemed to link a new word to an absent object based on the referential behaviour of a human. However, his performance in the second phase of Experiment 1 suggests that he might actually not have linked the word he heard with the specific object but instead – and most likely – linked the human’s general referential behaviour with the target object. This is interesting because, in the second study, there was some evidence that Paddy was actually able to do so when he had to inhibit any association of the human’s referential behaviour with an object in view. Paddy’s behaviour therefore indicates that he was to some extent able to use a human’s referential behaviour as a cue towards a specific, absent referent.

Human children use indexical, referential information which refers to absent entities, a skill absent in our closest living relatives, the chimpanzees [38]. Understanding referential cues toward absent entities is seen as a significant skill in the context of human language acquisition. (e.g., [22], [39]. It opens up the possibility of learning word-object relations outside the boundaries of spatiotemporal contiguity, therefore facilitating the learning of a nearly unlimited spectrum of word-object relations.

However, given the dogs’ limited skills in the studies presented here it is as yet unclear whether understanding a reference towards an absent referent is truly within dogs’ cognitive abilities. Even though Paddy showed some choice strategies, which suggests that he may have understood this concept, the question remains as to why he failed in forming object-label links when confronted with two objects at once. It could be that learning two labels at the same time is cognitively too demanding for a dog (see [40] for evidence that a label-trained dog can struggle with even easier versions of this experiment). It could also be that Paddy’s behaviour in the current task means that dogs correctly interpret referential behaviour as being *about* something but that they cannot link the spoken word to the object while doing so. Several studies have already shown that dogs are good at interpreting a human’s referential behaviour [33], [41]. Paddy’s behaviour could therefore be the first evidence to date that a dog attends to the referential aspects of humans’ communicative behaviour in situations in which reference is made to an absent object.

However, the question remains as to why the other three dogs struggled with all versions of the current studies and why the dogs performed so poorly overall in the different tasks. Kaminski et al. (2004) showed that the Border Collie Rico could learn a word-object link through exclusion and when no spatiotemporal contiguity between the word and the referent was given. This shows that this skill should be within the species’ range, although it could be that it is a skill that pushes the boundaries of what dogs are cognitively capable of, which is why major individual differences occur. What is more, the experimental settings of the current studies were probably especially challenging for the dogs given that these kinds of tasks are normally quite novel for dogs even if they have experience with objects being labelled. As reported by the owners of the different dogs, the usual procedure for learning word-object links is that the new label is constantly repeated while allowing the dog to play with the new object. Paddy’s behaviour does however leave room for the possibility that dogs can learn label-object relations through human referential communication and when no spatiotemporal contiguity is given. Future studies which are cognitively less demanding will need to investigate this further.

## References

[1] Pepperberg IM (1981) Functional vocalizations by an African grey parrot (Psittacus erithacus). Zeitschrift für Tierpsychologie 55: 139–160.

[2] Herman LM, Richards DG, Wolz JP (1984) Comprehension of sentences by bottlenosed dolphins. Cognition 16: 129–219. 6540652 10.1016/0010-0277(84)90003-9

[3] Savage-Rumbaugh S, McDonald K, Sevcik RA, Hopkins WD, Rubert E (1986) Spontaneous symbol acquisition and communicative use by pygmy chimpanzees (Pan paniscus). Journal of Experimental Psychology: General 115: 211–235. 2428917 10.1037//0096-3445.115.3.211

[4] Savage-Rumbaugh E (1986) Ape language: From conditioned response to symbol. New York, NY: Columbia University Press. xxv, 433 p.

[5] Schusterman RJ, Krieger K (1986) Artificial language comprehension and size transposition by a California sea lion (Zalophus californianus). Journal of Comparative Psychology 100: 348–355. 3802779

[6] Coppinger R, Coppinger L (2001) Dogs: A startling New Understanding of Canine origin, Behavior and Evolution. New York: Scribner.

[7] Hare B, Tomasello M (2005) Human-like social skills in dogs? Trends in Cognitive Sciences: 439–444. 10.1016/j.tics.2005.07.003 16061417

[8] Pilley J, Reid A (2011) Border collie comprehends object names as verbal referents. Behavioural Processes 86: 184–195. 21145379 10.1016/j.beproc.2010.11.007

[9] Kaminski J, Call J, Fischer J (2004) Word learning in a domestic dog: Evidence for “fast mapping”. Science 304: 1682–1683. 15192233 10.1126/science.1097859

[10] Carey S, Bartlett E (1978) Acquiring a single new word. Papers and Reports on Child Language Development 15: 17–29.

[11] Heibeck TH, Markman EM (1987) Word learning in children: An examination of fast mapping. Child Development 58: 1021–1034. 3608655

[12] Markson L, Bloom P (1997) Evidence against a dedicated system for word learning in children. Nature 385: 813–815. 9039912 10.1038/385813a0

[13] Bloom P (2004) Can a dog learn a word? Science 304: 1605–1606. 15192205 10.1126/science.1099899

[14] Markman EM, Abelev M (2004) Word learning in dogs? Trends in Cognitive Sciences 8: 479–481. 15491899 10.1016/j.tics.2004.09.007

[15] Baldwin DA (1991) Infants’ contribution to the achievement of joint reference. Child Development 62: 875–890. 1756664

[16] Baldwin DA (1993) Infants’ ability to consult the speaker for clues to word reference. Journal of Child Language 20: 395–418. 8376476 10.1017/s0305000900008345

[17] Tomasello M, Barton ME (1994) Learning words in nonostensive contexts. Developmental Psychology 30: 639–650.

[18] Baldwin DA, Markman EM, Bill B, Desjardins RN, Irwin JM (1996) Infants’ reliance on a social criterion for establishing word-object relations. Child Development 67: 3135–3153. 9071774

[19] Tomasello M, Barton M (1994) Learning words in non-ostensive contexts. Developmental Psychology 16: 495–552.

[20] Tomasello M, Strosberg R, Akhtar N (1996) Eighteen-month-old children learn words in non-ostensive contexts. Journal of Child Language 23: 157–176. 8733565 10.1017/s0305000900010138

[21] Tomasello M (2001) Perceiving intentions and learning words in the second year of life. Tomasello, Michael (Ed); Bates, Elizabeth (Ed) (2001) Language development: The essential readings Essential readings in developmental psychology (pp 111–128) vii, 375pp.

[22] Tomasello M (2008) Origins of human communication. Cambridge, MA: The MIT Press.

[23] Bates E, Benigni L, Bretherton I, Camaioni L, Volterra V (1979) The emergence of symbols: cognition and communication in infancy. New York: Academic Press.

[24] Wobber V, Kaminski J (2011) What do dogs understand about human communicative signals? A novel synthesis. Dogs: biology, behavior, and health disorders. new York: Nova Press,. 93–110.

[25] Bräuer J, Kaminski J, Riedel J, Call J, Tomasello M (2006) Making Inferences About the Location of Hidden Food: Social Dog, Causal Ape. Journal of Comparative Psychology 120: 38–47. 16551163 10.1037/0735-7036.120.1.38

[26] Hare B, Brown M, Williamson C, Tomasello M (2002) The domestication of social cognition in dogs. Science 298: 1634–1636. 12446914 10.1126/science.1072702

[27] Kirchhofer KC, Zimmermann F, Kaminski J, Tomasello M (2012) Dogs (Canis familiaris), but not chimpanzees (Pan troglodytes), understand imperative pointing. PLOs one 7: e30913. 22347411 10.1371/journal.pone.0030913 PMC3275610

[28] Miklosi A, Kubinyi E, Topal J, Gacsi M, Viranyi Z, et al (2003) A simple reason for a big difference: Wolves do not look back at humans, but dogs do. Current Biology [print] 13: 763–766. 10.1016/s0960-9822(03)00263-x 12725735

[29] Riedel J, Schumann K, Kaminski J, Call J, Tomasello M (2008) The early ontogeny of human-dog communication. Animal Behaviour 75: 1003–1014.

[30] Virányi Z, Gácsi M, Kubinyi E, Topál J, Belényi B, et al (2008) Comprehension of human pointing gestures in young human-reared wolves (Canis lupus) and dogs (Canis familiaris). Animal Cognition 11: 373–387. 18183437 10.1007/s10071-007-0127-y

[31] Udell M, Dorey N, Wynne C (2008) Wolves outperform dogs in following human social cues. Animal Behaviour 76: 1767–1773.

[32] Hare B, Rosati A, Kaminski J, Bräuer J, Call J, et al (2010) The domestication hypothesis for dogs’ skills with human communication: a response to Udell et al.(2008) and Wynne et al. (2008). Animal Behaviour 79: e1–e6.

[33] Kaminski J, Schulz L, Tomasello M (2012) How dogs know when communication is intended for them. Developmental Science. 10.1111/j.1467-7687.2011.01120.x 22356178

[34] Kaminski J, Tempelmann S, Call J, Tomasello M (2009) Domestic dogs comprehend human communication with iconic signs. Developmental Science 12: 831–837. 19840038 10.1111/j.1467-7687.2009.00815.x

[35] Kaminski J, Fischer J, Call J, Cognition A (2008) Prospective object search in dogs: mixed evidence for knowledge of What and Where. Animal Cognition 11: 367–371. 18060437 10.1007/s10071-007-0124-1 PMC2757617

[36] Grassmann S, Kaminski J, Tomasello M (2012) How two word-trained dogs integrate pointing and naming. Animal Cognition: 1–9. 10.1007/s10071-012-0494-x PMC3377900 22526689

[37] Manly B (1997) Randomization, Bootstrap and Monte Carlo Methods in Biology. London: Chapman and Hall.

[38] Liszkowski U, Schaefer M, Carpenter M, Tomasello M (2009) Prelinguistic infants, but not chimpanzees, communicate about absent entities. Psychological Science 20: 654. 19476595 10.1111/j.1467-9280.2009.02346.x

[39] Liszkowski U, Carpenter M, Tomasello M (2007) Reference and attitude in infant pointing. Journal of Child Language 34: 1–20. 17340936 10.1017/s0305000906007689

[40] Griebel U, Oller D (2012) Vocabulary Learning in a Yorkshire Terrier: Slow Mapping of Spoken Words. PLOs one 7: e30182. 22363421 10.1371/journal.pone.0030182 PMC3281824

[41] Téglás E, Gergely A, Kupán K, Miklósi Á, Topál J (2012) Dogs’ gaze following is tuned to human communicative signals. Current Biology. 10.1016/j.cub.2011.12.018 22226744

